# ß-catenin signaling is required for RAS-driven thyroid cancer through PI3K activation

**DOI:** 10.18632/oncotarget.10356

**Published:** 2016-06-30

**Authors:** Ana Sastre-Perona, Garcilaso Riesco-Eizaguirre, Miguel A. Zaballos, Pilar Santisteban

**Affiliations:** ^1^ Instituto de Investigaciones Biomédicas “Alberto Sols”, Consejo Superior de Investigaciones Científicas (CSIC) y Universidad Autónoma de Madrid (UAM), Madrid, Spain; ^2^ Servicio de Endocrinología, Hospital Universitario de Móstoles, Madrid, Spain

**Keywords:** ß-catenin, PI3K, RAS, thyroid cancer

## Abstract

Mutations in ß-catenin are traditionally described as late events in thyroid cancer progression. However, the functional implications of ß-catenin dysregulation in the context of tumor initiating events remain unclear. The aim of this work was to investigate whether the two main oncogenic drivers in thyroid cancer, RAS and BRAF, could activate the Wnt/ß-catenin pathway. Expression of HRAS^V12^ but not BRAF^V600E^ in thyroid cells induced ß-catenin nuclear localization, increased ß-catenin-dependent transcriptional activity and inhibited GSK3ß. In a panel of human thyroid cancer cell lines representative of the main genetic events in thyroid cancer, ß-catenin activation was highly dependent on PI3K/AKT activity through its phosphorylation at S552, but not on MAPK. Silencing of ß-catenin expression in cell lines led to a dramatic reduction in proliferation due to an induction of senescence, which was concordant with a reduction in tumor size in nude mice. Moreover, ß-catenin silencing suppressed the expression of EMT-related genes and reduced the invasive capacity of the tumor cells. In conclusion, this work demonstrates that RAS-driven tumors induce PI3K/AKT-dependent ß-catenin activation.

## INTRODUCTION

The most recent advances in thyroid cancer research emerge from an increased understanding of the mechanisms that control thyroid cell proliferation and differentiation, and the associated signal transduction pathways [1]. The current model of thyroid cancer development assumes that accumulation of mutations in tumor cells drives progression through a dedifferentiation process that leads initially to well-differentiated carcinomas, such as papillary (PTC) and follicular (FTC) thyroid carcinoma, and progresses to poorly differentiated (PDC) and undifferentiated or anaplastic (ATC) thyroid carcinomas [2, 3]. Initiation and progression of thyroid cancer comprises multiple genetic alterations, of which mutations leading to the activation of the MAPK and PI3K/AKT signaling pathways are the most studied. In PTC, MAPK activation is crucial for tumor initiation. Characterized defects in this pathway include mutations in the intracellular signal transducers RAS and BRAF, and rearrangements in the cell-membrane tyrosine kinase receptors RET (RET/PTC); these mutations are mutually exclusive [4–8]. In FTC, PI3K/AKT activation is thought to arise from RAS mutations, inactivating mutations in the PTEN or RASL1 tumor suppressor genes, or by activating mutations in PIK3CA and AKT1 [9–11]. Thyroid cancer progression to PDC and ATC involves additional mutations that stimulate other cell signaling pathways, such as p53 and Wnt/ß-catenin [12]. This latter pathway is constitutively activated in 50% of PDC and ATC due to mutations in ß-catenin and Axin genes [13–15]. These mutations impair normal ß-catenin degradation, resulting in its accumulation in the cytoplasm and nucleus of tumor cells, and activation of target genes involved in biological functions essential for carcinogenesis [15–19].

Although no mutations in ß-catenin or Axin have been identified in PTC or FTC, ß-catenin dysregulation, particularly the reduction of ß-catenin plasma membrane levels as well as its aberrant nuclear localization, closely parallel loss of tumor differentiation and poor prognosis in human samples [14].

These data suggest that additional mechanisms, including post-translational modifications, could be responsible for ß -catenin mislocalization in thyroid tumors in the absence of mutations in Wnt components. The PI3K/AKT pathway is traditionally linked to activation of the Wnt canonical pathway through the inhibition of GSK3ß [20], which is a critical factor of the ß-catenin destruction complex. GSK3ß phosphorylates ß-catenin at S33/37/T41, resulting in its ubiquitination and degradation through the proteasome. Recent evidence has shown that Akt can directly phosphorylate ß-catenin at S552, leading to its stabilization and increasing its transcriptional activity [21, 22]. Moreover, PI3K signaling has been shown to control ß-catenin activity in normal thyroid gland in response to TSH and IGFI [23]. In addition, the MAPK pathway has been described to activate Wnt signaling through ERK-mediated inhibition of GSK3ß [24].

In an *in vitro* model of thyroid cancer, oncogenic RET/PTC, present only in PTC, induces ß-catenin stabilization and nuclear accumulation by a Wnt-independent mechanism involving activation of PI3K/AKT and MAPK signaling pathways [25–27]. However, the consequences on ß-catenin signaling in genetic contexts other than RET/PTC are unknown. Therefore, the aim of this work was to investigate whether other oncogenic drivers, such as RAS, BRAF or loss of PTEN, could activate the Wnt/ß-catenin pathway and participate in thyroid carcinogenesis.

Here we show that HRAS, but not BRAF, induces ß-catenin activation, unveiling a novel mechanism of ß-catenin stabilization in thyroid tumor cells contingent on AKT activity. These findings strongly support the functional participation of ß-catenin in cell proliferation and epithelial-mesenchymal transition (EMT), and suggest that it could be a potential therapeutic target for treatment of thyroid cancer.

## RESULTS

### RAS but not BRAF induces Wnt/ß-catenin activation in thyroid cells

**Figure 1:**
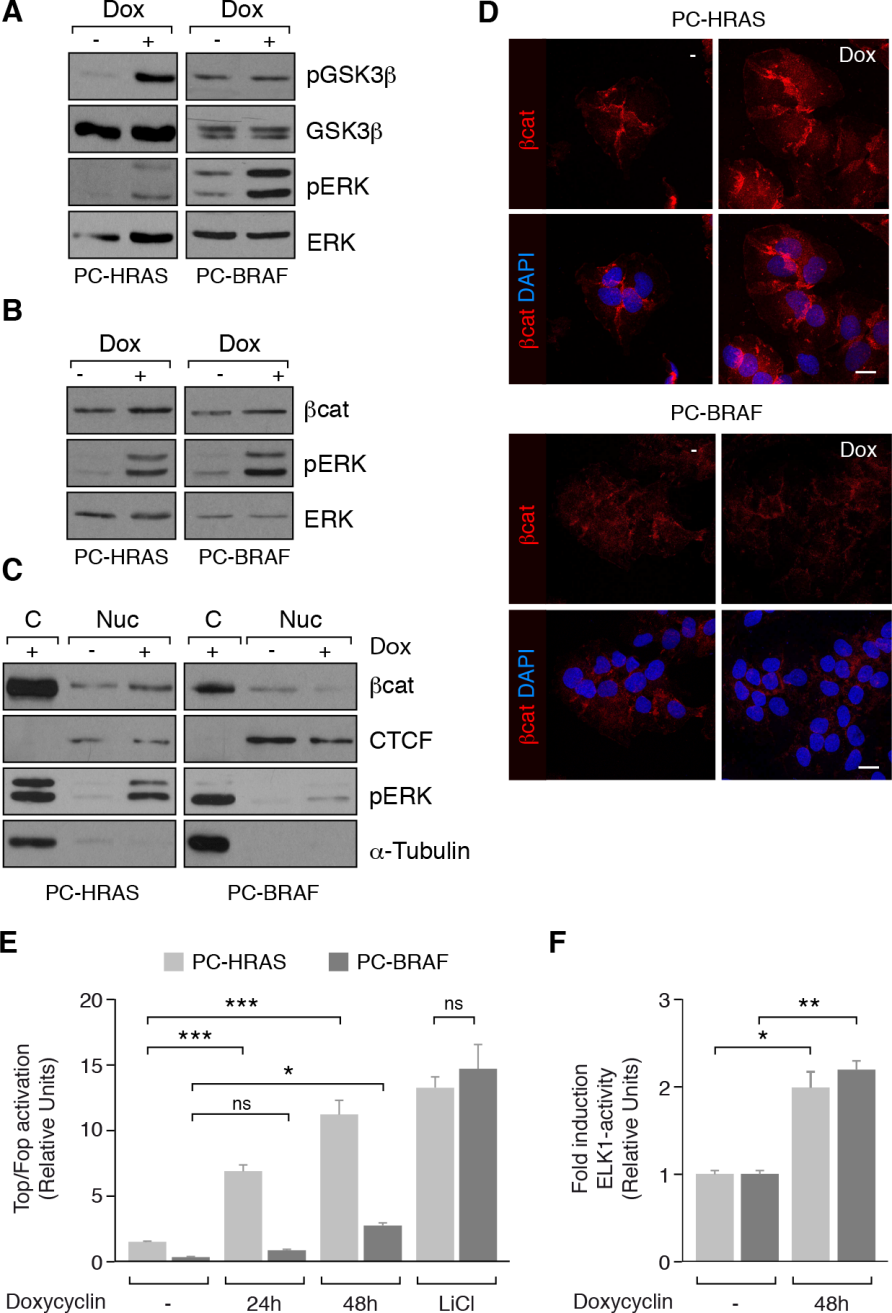
Wnt/ß-catenin activation in PCCl3 cells conditionally expressing HRAS^V12^ (PC-HRAS) or BRAF^V600E^ (PC-BRAF) PC-HRAS and PC-BRAF cells were starved for 48 h and then treated with doxycycline for the times indicated. (**A** and **B**). Total proteins extracts were analyzed by western blot for the detection of p-GSK3ß (panel A) and ß-catenin (ßcat) (panel B). (**C**) Nuclear (Nuc) and cytoplasmic (C) protein extracts were analyzed by western blot for the detection of ß-catenin. CTCF and ß-tubulin were used as nuclear and cytoplasmic loading controls, respectively. (**D**) Cells were grown on cover-slips, fixed and stained with a ß-catenin antibody (red). Nuclei were stained with DAPI (blue). Scale bar 10 μm. (**E**) ß-catenin transcriptional activity was measured in cells transfected with Super8x TopFlash (Top) or Super8x FopFlash (Fop) vectors after 24 and 48 h of doxycycline treatment or 48 h of LiCl treatment. Data show fold induction of the Top/Fop ratio with respect to non-treated cells (−). (**F**) ELK-1 activation was measured in cells transfected with the reporter vector pGE51-luc, encoding the transactivation domain of Elk-1 (amino acids 307 to 428) fused to the GAL4 DNA-binding domain, and a pRL-CMV Renilla construct, after 48 h in the presence (+) or absence (−) of doxycycline. Data show fold induction of ELK-1 activation relative to Renilla levels. Values represent means ± SEM (*n* = 3). *p < 0.05; **p < 0.01; *** *p* < 0.001.

We investigated whether the Wnt/ß-catenin pathway was active in the earliest steps of thyroid tumorigenesis driven by RAS and BRAF, the two main oncogenes in thyroid cancer [28]. To do this, we used rat thyroid-derived PCCl3 cells conditionally expressing HRAS^V12^ (PC-HRAS) or BRAF^V600E^ (PC-BRAF) after doxycycline treatment. As ß-catenin stabilization is due in part to GSK3ß inhibition, we examined GSK3ß phosphorylation at Ser9. Doxycycline treatment for 48 h increased GSK3ß levels in PC-HRAS cells but not in PC-BRAF cells, indicating that HRAS, but not BRAF, induced GSK3ß inhibition (Figure 1A). To assess whether this inhibition modified ß-catenin stabilization and its nuclear localization, we analyzed ß-catenin expression in total, cytoplasmic and nuclear extracts from PC-HRAS and PC-BRAF cells treated or not with doxycycline. Whereas both HRAS and BRAF oncogenes induced a minor increase in total ß-catenin levels (Figure 1B), only HRAS expression increased nuclear ß-catenin expression (Figure 1C). These findings were confirmed by immunocytochemistry and confocal imaging (Figure 1D). To test whether ß-catenin nuclear expression increased its transcriptional activity, PC-HRAS and PC-BRAF cells were transfected with the artificial Top/Fop promoter, which contains several ß-catenin/TCF binding sites in tandem, and luciferase activity was measured. Cells were treated with LiCl as a positive control of ß-catenin transcriptional activation. Expression of HRAS resulted in a robust and time-dependent increase in luciferase activity, reaching more than 10-fold at 48 h. By contrast, BRAF expression resulted in a minor increase (2-fold) in luciferase activity at 48 h after transfection (Figure 1E). To confirm that the reduced ability of BRAF to activate Top/Fop was not because of an overall reduced output of BRAF with respect to HRAS cells, we measured the ability of both oncogenes to activate the ERK effector ELK1. Expression of BRAF and HRAS induced the activation of ELK1 to a similar level (Figure 1F). These results show that HRAS, unlike BRAF, induces strong ß-catenin stabilization and activation in thyroid cells.

### PI3K stimulates ß-catenin expression and activity in human thyroid tumor cells, which is dependent on its phosphorylation at Ser 552

**Figure 2:**
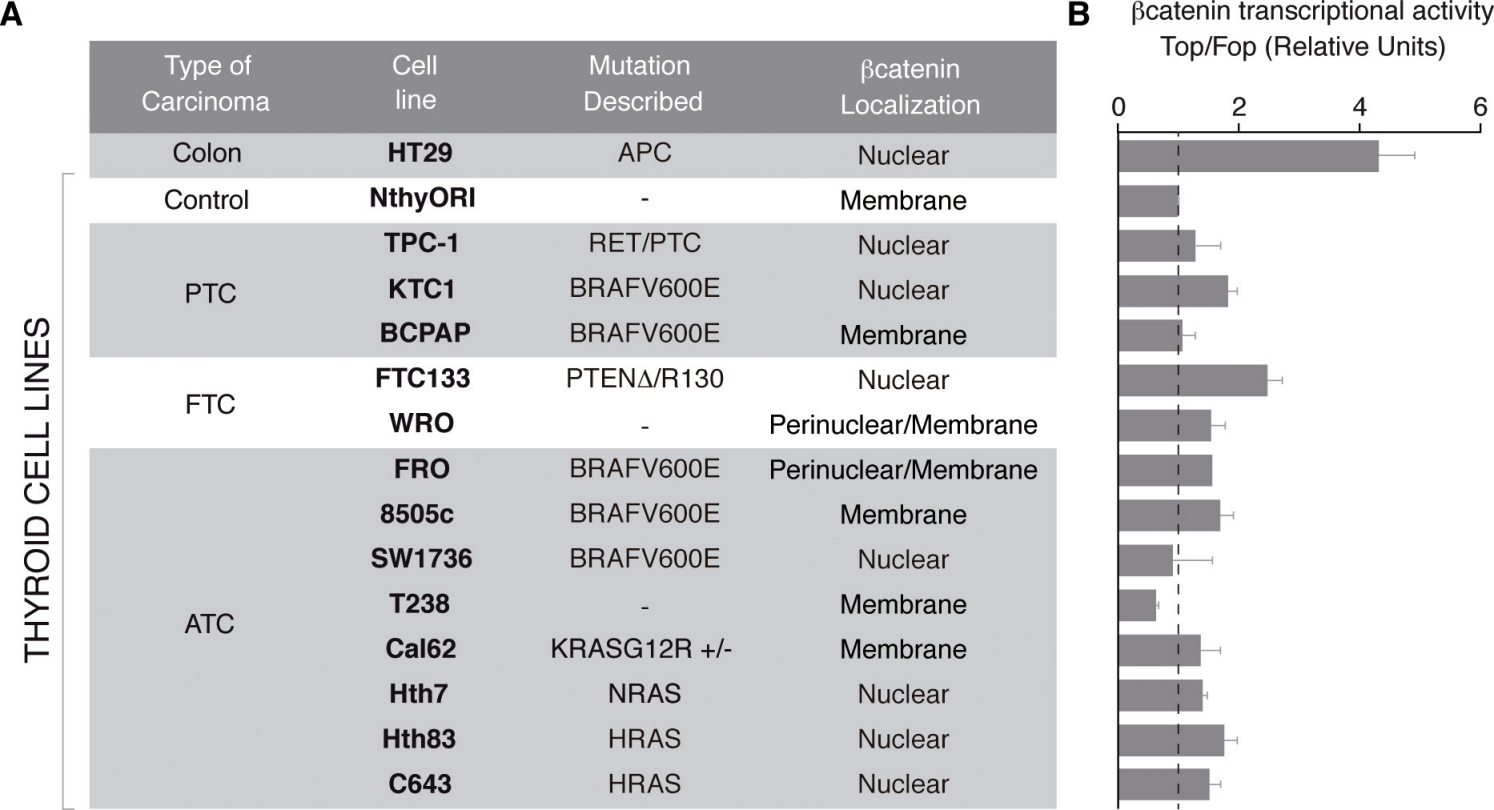
ß-catenin subcellular localization and transcriptional activity in a panel of human thyroid cancer cell lines (**A**) Table summarizing classification, mutations described and ß-catenin localization in different thyroid cell lines. ß-catenin localization was analyzed by immunocytochemistry and confocal imaging as shown in Supplementary Figure 1. (**B)** ß-catenin transcriptional activity was measured in Super8x (Top) or Super8x FopFlash vector-transfected cells. Data show fold induction of the Top/Fop ratio with respect to non-treated cells (−). Values represent means ± SEM (*n* = 3).

To assess the status of Wnt/ß-catenin signaling in human thyroid cancer, we surveyed ß-catenin subcellular localization and transcriptional activity in a panel of human thyroid cell lines representative of the main histologic types, and harboring the main genetic mutations of thyroid cancer (Figure 2). Human HT29 colon carcinoma cells carrying the APC mutation [29] and Nthy-ori 3–1 immortalized normal epithelial human thyroid cells [30] served as positive and negative controls, respectively. To assess the subcellular localization of ß-catenin, we performed immunocytochemistry and confocal imaging (see Supplementary Figure 1 and summary in Figure 2A). According to the subcellular localization of ß-catenin, we found three types of cells. One type expressed ß-catenin exclusively in the plasma membrane (8505c, T238, Cal62 and Nthy-ori 3–1). A second type expressed ß-catenin both in the plasma membrane and in the cytoplasm, with a marked perinuclear accumulation (WRO and FRO). A third type of cell expressed ß-catenin exclusively in the nucleus (FTC133, SW1736, Hth7, Hth83, C643, TPC1 and KTC1), comparable with HT-29 colon carcinoma. Of note, the majority of RAS mutated cells (3 out of 4) presented ß-catenin nuclear localization.

We next assessed ß-catenin transcriptional activity (Figure 2B). Unexpectedly, no correlation was found between the subcellular distribution of ß-catenin and its transcriptional activity (Figure 2A, 2B). Generally, ß-catenin transcriptional activity in thyroid cancer cell lines was lower (~2-fold) than in HT-29 positive cells (5-fold). Notably, FCT133 cells, which harbor a PTEN deletion, had the greatest ß-catenin transcriptional activity, up to > 3-fold.

**Figure 3:**
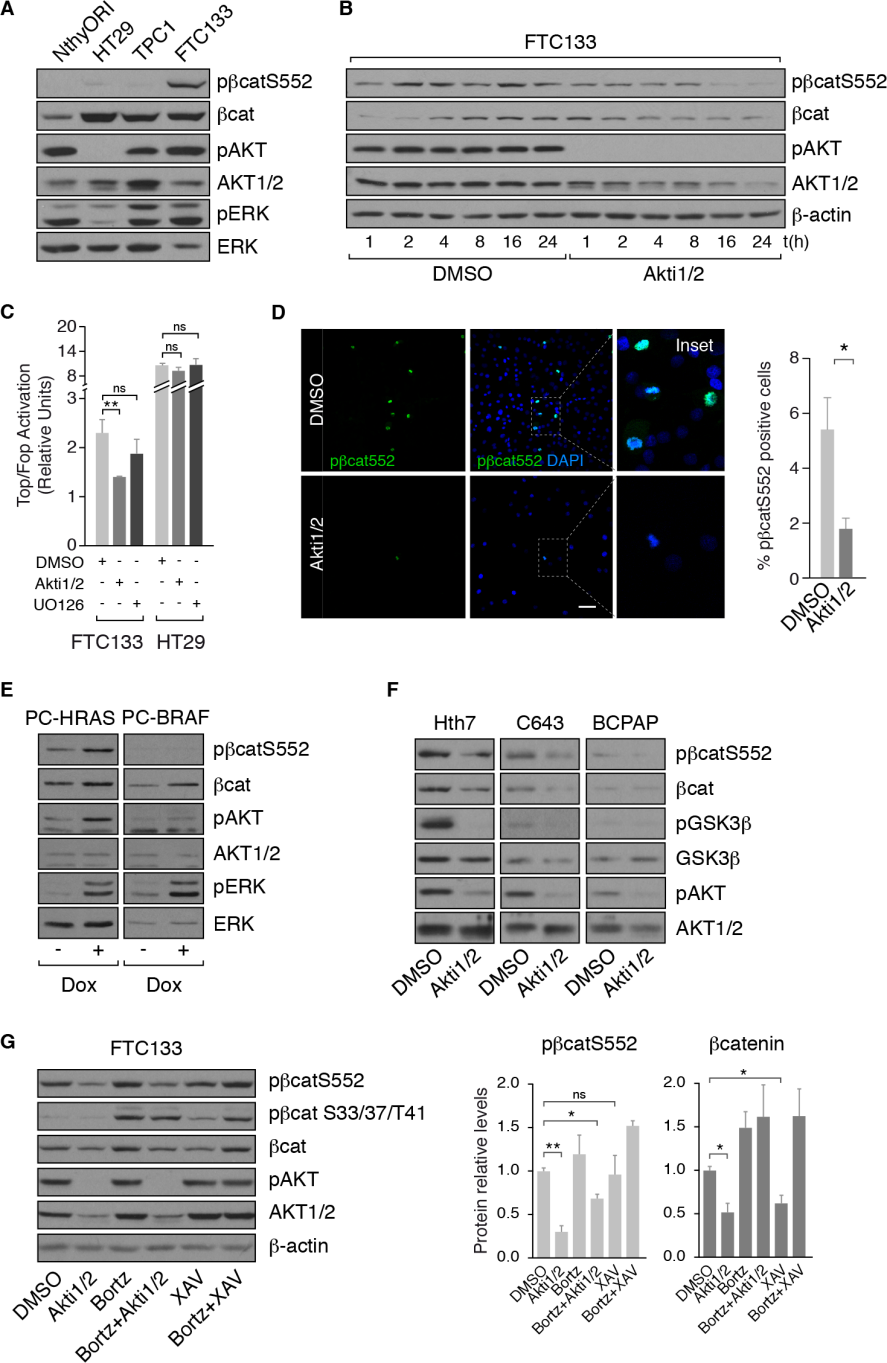
PI3K/AKT signaling controls ß-catenin expression through its phosphorylation at serine 552 (**A, B)** ß-catenin and pß-cateninS552 expression levels were analyzed by western blot in the indicated cell lines and in FTC133 cells treated with DMSO or with Akti1/2 for the specified times. (**C**) ß-catenin transcriptional activity was analyzed in FTC133 and HT29 cells after treatment with DMSO, Akti1/2 or UO126 for 24 h. Data show fold induction of the Top/Fop ratio with respect to non-treated cells (−). Values represent means ± SEM (*n* = 3). *p < 0.05; **p < 0.01; ***p < 0.001. (**D**) FTC133 cells were grown on cover-slips, treated with DMSO or Akti1/2 for 24 h, fixed and stained for pß-cateninS552 (green). Nuclei were stained with DAPI (blue). Scale bar 25 μm. The insets show higher magnifications of the regions marked by a square. (**E**) PC-HRAS and PC-BRAF were starved for 48 h and then treated with doxycycline for 48 h. Total protein extracts were analyzed by western blot for pß-cateninS552. (**F**) The indicated cell lines were treated with DMSO or Akti1/2 and total protein extracts were analyzed by western blot for the detection of ß-catenin and pß-cateninS552. (**G)** Left panel: FTC133 cells were pre-treated for 1 h with bortezomib (Bortz) and then treated with DMSO, Akti1/2 or XAV939 (XAV) for 8 h. Total protein extracts were analyzed by western blot for the detection of pß-cateninS552 and pß-cateninS33/37/T41. Right panel: quantification of protein levels normalized to ß-actin. Values represent means ± SEM (*n* = 3). *p < 0.05; **p < 0.01; ns not significant.

Since FTC133 exhibited robust nuclear ß-catenin expression and had the highest transcriptional activity, we used this cell line to examine the mechanisms of activation of ß-catenin signaling. FTC133 cells derive from a follicular thyroid carcinoma, harbor a PTEN deletion and, accordingly, exhibit a high PI3K activity as shown by their high levels of AKT phosphorylation (Figure 3A). Western blotting of total cell extracts revealed high expression of total ß-catenin in FTC133 cells (Figure 3A), which was comparable to its expression in HT-29 and TPC-1 cells, two cell types exhibiting ß-catenin activation [25, 31]. Interestingly, FTC133 also showed high levels of ß-catenin phosphorylated at Ser552, which promotes ß-cateninentry into the nucleus and increases its transcriptional activity (Figure 3A) [21, 32]. We previously reported that TSH and IGF1, through PKA and AKT, respectively, phosphorylate ß-catenin at Ser552 in normal differentiated PCCl3 thyroid cells [23]. In thyroid tumor cells, the cAMP/PKA pathway is impaired due to oncogene activation [33, 34]. To determine whether ß-catenin phosphorylation was a direct consequence of PI3K/AKT activation, FTC133 cells were treated with Akti1/2, a specific AKT inhibitor, and phospho-Ser552 ß-catenin levels were measured. AKT inhibition decreased phospho-Ser552 ß-catenin expression (Figure 3B) and subsequent ß-catenin transcriptional activity (Figure 3C). By contrast, MAPK/MEK inhibition failed to suppress ß-catenin transcriptional activity in FTC133 cells (Figure 3C). In HT-29 cells, which do not have a constitutively active PI3K pathway (Figure 3A), the transcriptional activity of ß-catenin was unaffected by AKT inhibition (Figure 3C). Consistent with these results, confocal imaging showed that phospho-Ser552 ß-catenin was localized in the nuclei of FTC133 cells and its presence was contingent on AKT activity (Figure 3D).

In light of these findings and since BRAF^V600E^ oncogene activates MAPK pathway and HRAS^V12^ oncogene activates both MAPK and PI3K pathways, we evaluated phospho-Ser552 ß-catenin levels in PC-BRAF and PC-HRAS cells. HRAS but not BRAF activation increased phospho-Ser552 ß-catenin levels in PCCl3 cells (Figure 3E). In agreement with this finding, whereas RAS mutant human cell lines (Hth7 and C643) expressed phospho-Ser552 ß-catenin that was dependent on AKT activity (Figure 3F), the BRAF mutant cell line BCPAP did not. These data strengthen the notion that PI3K/AKT stabilizes ß-catenin in thyroid cancer.

PI3K/AKT-mediated phosphorylation of ß-catenin at Ser552, unlike phosphorylation at the N-terminus, increases its stabilization, leading to its accumulation and translocation into the nucleus [22]. To test if this mechanism was indeed responsible for ß-catenin stability in FTC133 cells, we used bortezomib to inhibit proteasome activity and co-treated cells with/without Akti1/2 to inhibit AKT activity. Western blotting showed that the degradation of total ß-catenin after AKT inhibition was impaired with bortezomib and, thus, dependent on proteasome activity (Figure 3G). This result shows that phosphorylation at Ser552 is essential for AKT-induced ß-catenin stabilization.

Finally, to examine whether the canonical Wnt pathway participated in PI3K/AKT-mediated stabilization of ß-catenin, we inhibited Wnt signaling in FTC133 cells with XAV939 [35]. Inhibition had no effect on phospho-Ser552 ß-catenin levels, suggesting that PI3K/AKT-mediated stabilization of ß-catenin is independent of Wnt. However, inhibition of Wnt signaling decreased total ß-catenin expression and increased phospho-Ser33/37/T41-ß-catenin, an N-terminal phosphorylation that promotes ß-catenin degradation (Figure 3G) [35]. By contrast, inhibition of AKT had no effect on phosphorylation at the N-terminus. Collectively, these results suggest that PI3K/AKT and Wnt induce ß-catenin stabilization through independent mechanisms in thyroid cancer cells that is dependent on its phosphorylation status, similar to that described in normal thyroid cells [23].

Overall, these data support a critical function for PI3K/AKT signaling, but not MAPK signaling, in ß-catenin stabilization in thyroid tumor cells. This PI3K/AKT-controlled stabilization of ß-catenin occurs through its phosphorylation at Ser552 and is independent of Wnt.

### ß-catenin silencing inhibits proliferation and induces senescence in human thyroid tumor cells

**Figure 4:**
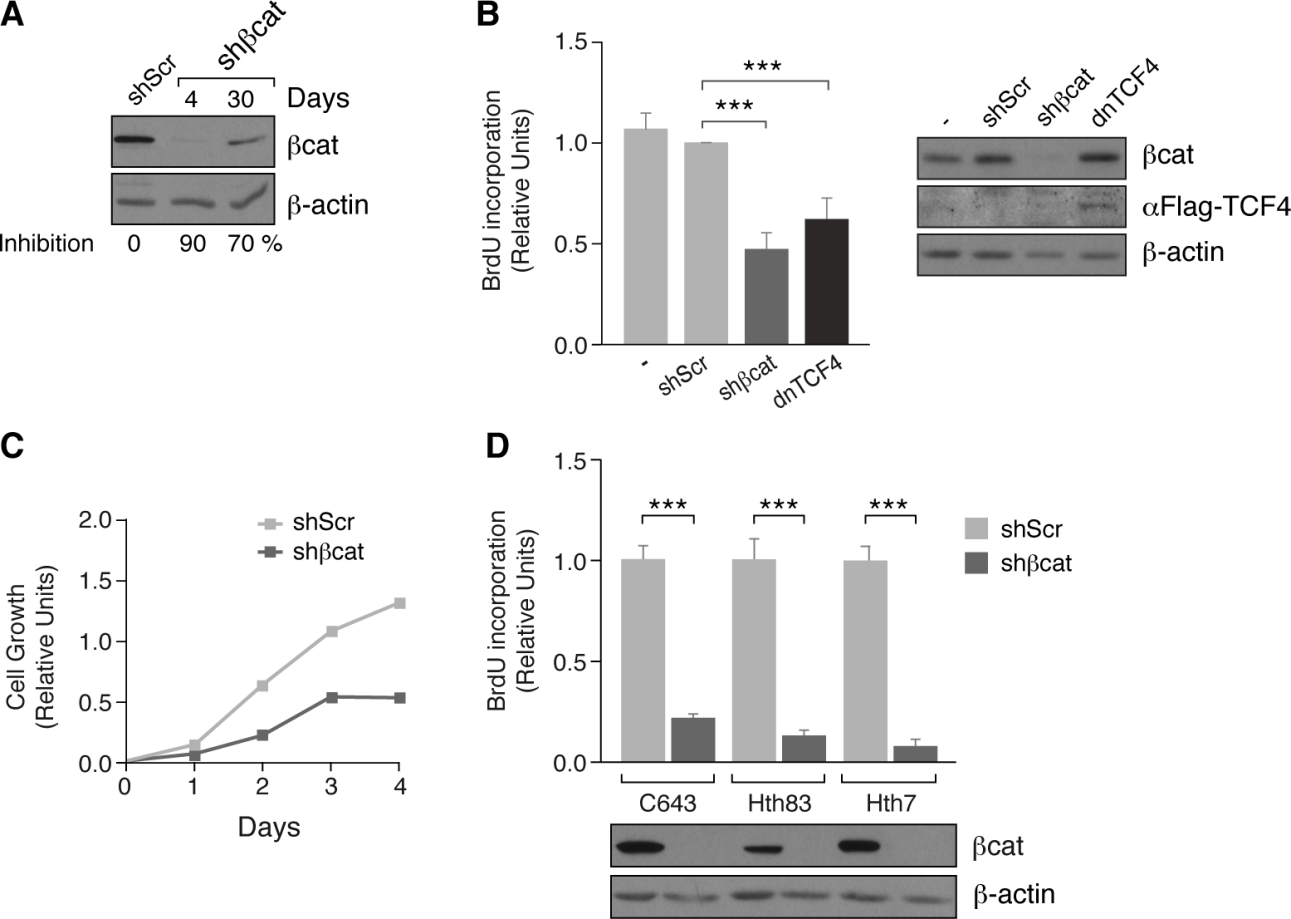
ß-catenin inhibition decreases proliferation in human tumoral thyroid cells (**A)** FTC133 cells were infected with control (shScr) or ß-catenin (shßcat) lentivirus shRNAs. Total protein extracts were obtained at the indicated times and analyzed by western blot for ß-catenin expression. (**B**) Left panel: FTC133 cells were infected with control (shScr) or ß-catenin (shßcat) lentivirus shRNAs or a dominant-negative form of TCF4 [dnTCF4] and BrdU incorporation was calculated. Data show BrdU incorporation relative to that of cells infected with the vehicle vector (shScr). Values represent means ± SEM (*n* = 3). ***p < 0.001. Right panel: western blot analyis of TCF4 expression (**C**) FTC133 cells were infected with control (shScr) or ß-catenin (shßcat) lentivirus shRNAs and cell growth was estimated relative to the density at day 0. Values represent means ± SEM (*n* = 3). (**D**) Cell lines were infected with control (shScr) or ß-catenin (shßcat) lentivirus shRNAs and BrdU incorporation was calculated. Data show BrdU incorporation relative to that of the cells infected with the vehicle vector (shScr). ***p < 0.001.

To study the role of ß-catenin in thyroid tumor cell proliferation, we used a lentivirus expressing a short hairpin RNA (shRNA) to stably silence ß-catenin in FTC133 cells (Figure 4A). As expected, the level of DNA synthesis in ß-catenin shRNA cells was decreased by 50% relative to scrambled shRNA-transfected cells (Figure 4B). This was paralleled by a decrease in proliferation of ß-catenin shRNA cells (Figure 4C). Similarly, a significant decrease in DNA synthesis was detected in ß-catenin shRNA-transfected RAS mutant thyroid cancer cell lines (C643, Hth83, and Hth7) (Figure 4D). To confirm that this effect was due to ß-catenin transcriptional activity and not because of its role in cell adhesion, we stably expressed a dominant-negative form of FLAG-tagged TCF4 (*Transcription factor 7-like 2 gene*) in FTC133 cells, which does not bind ß-catenin [36], and measured DNA synthesis. BrdU incorporation was significantly lower in TCF4-expressing cells than in controls (Figure 4B), suggesting that FTC133 cell proliferation required the transcriptional activity of ß-catenin independent of its involvement in cell adhesion.

**Figure 5:**
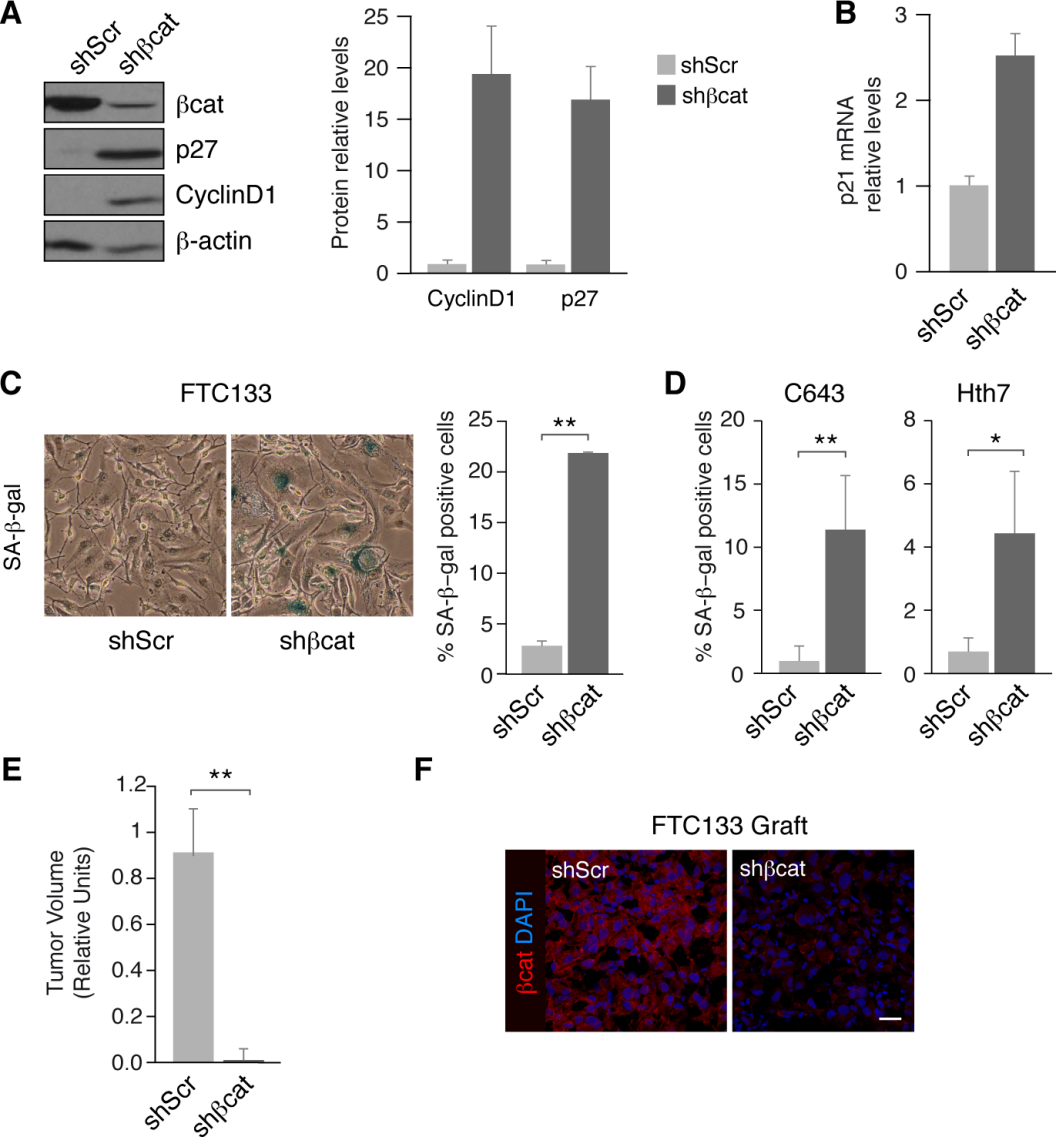
ß-catenin inhibition induces cell senescence and reduces tumor growth in human tumoral thyroid cells (**A**) FTC133 cells were infected with control (shScr) or ß-catenin (shßcat) lentivirus shRNAs. Left panel: total protein extracts were obtained and analyzed by western blot for the detection of ß-catenin, cyclinD1 and p27. Right panel: quantification of cyclinD1 and p27 protein levels normalized to β-actin. Values represent means ± SEM (*n* = 3). (**B**) p21 mRNA expression levels were analyzed in FTC133 shScr and shßcat-infected cells. (**C**) FTC133 cells were infected with control (shScr) or ß-catenin (shßcat) lentivirus shRNAs and analyzed for SA-ßgal-positivity. Left panel: representative ßgal-staining. Right panel: percentage of SA-ßgal positive cells per field. Values represent means ± SEM (*n* = 3). **p < 0.01. (**D**) C643 and Hth83 cells were infected with control (shScr) or ß-catenin (shßcat) lentivirus shRNAs and analyzed for SA-ßgal-positivity. Percentage of SA-ßgal positive cells per field. Values represent means ± SEM (*n* = 3). **p < 0.01. (**E**) FTC133 control (shScr) or shßcat cells were injected in NOD-SCID mice and the tumor volume was measured at the end of the experiment. Values represent means ± SEM (*n* = 3). **p < 0.01. (**F**) FTC133 grafts from control (shScr) or shßcat cells were stained with a ß-catenin antibody (red). Nuclei were stained with DAPI (blue). Scale bar 25 μm.

Since cyclinD1 is a direct downstream target of ß-catenin activation, we sought to study its function in cell proliferation. Unexpectedly, silencing of ß-catenin markedly increased cyclin D1 levels (Figure 5A) and also the levels of the cell cycle inhibitors p27^kip1^ and p21 (Figure 5A, 5B). This surprising result led us to hypothesize that the high expression of cyclin D1 and cell cycle inhibitors were in fact indicators of senescence. Indeed, previous studies have demonstrated that upregulation of cyclinD1 occurs in senescent cells [37–39]. Thus, we determined ß-galactosidase (SA-ß-gal) activity as a metric of senescence. Silencing of ß-catenin resulted in a significant increase in the percentage of SA-ß-gal-positive cells relative to non-silenced controls (Figure 5C). Comparable results were obtained in ß-catenin-silenced C643 and Hth7 cells (Figure 5D), demonstrating that ß-catenin silencing also increases senescence in RAS mutant human thyroid cell lines.

To verify this finding *in vivo*, we injected control or ß-catenin shRNA FTC133 cells subcutaneously into immunocompromised NOD-SCID mice and monitored the development of xenografts. Xenograft tumor size was dramatically reduced in ß-catenin-silenced tumors with respect to the control tumors (Figure 5E). Importantly, the expression levels of ß-catenin remained inhibited in xenografts of ß-catenin-silenced cells compared with the control cells (Figure 5F). Collectively, these results strongly suggest that inhibition of ß-catenin activity decreases thyroid tumor cell proliferation by promoting cell cycle arrest and inducing senescence.

### ß-catenin silencing induces E-cadherin expression and diminishes EMT markers expression and tumor cell invasion

**Figure 6:**
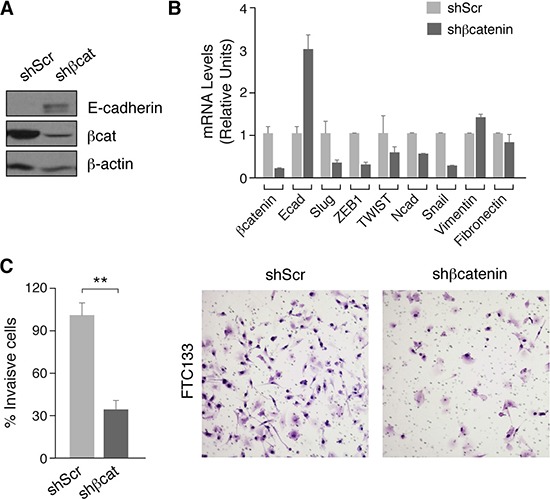
ß-catenin silencing decreases expression of EMT markers and inhibits cell migration of human thyroid tumoral cells (**A**) FTC133 cells were infected with control (shScr) or ß-catenin (shßcat) lentivirus shRNAs. Total protein extracts were analyzed by western blot for the detection of ß-catenin and E-cadherin. (**B**) Expression of selected EMT genes. Data show the relative expression levels compared with the control cells. (**C**) The invasive capacity of FTC133 control or shß-catenin cells was analyzed in Matrigel-coated Transwells. Left panel: number of cells invading the lower chamber after 24 h. A total of 10 fields for each cell line were quantified. Values represent means ± SEM (*n* = 3).**p < 0.01. Right panel: representative images of Transwell filters.

A feature common to epithelial tumor cells is the acquisition of a mesenchymal phenotype characterized by an increased invasion capacity. Epithelial-mesenchymal transition (EMT) is induced by many signaling pathways including Wnt/ß-catenin and is characterized by the loss of E-cadherin expression through activation of transcriptional repressors such as ß-catenin/TCF, Snail, Slug, TWIST or ZEB1/2 [40]. Inhibition of E-cadherin expression has been described in BRAF-driven thyroid tumors in which the MAPK pathway is activated [41], but little is known about the mechanisms involved in E-cadherin repression in RAS-driven tumors where PI3K/AKT is activated. Given our findings of PI3K regulation of ß-catenin activity, we examined its role on E-cadherin expression in FTC133 thyroid cells. Silencing of ß-catenin resulted in an increase in E-cadherin protein and mRNA expression (Figure 6A, 6B). This was accompanied by a decrease in the expression of transcription factors Slug and Twist, which are also controlled by ß-catenin/TCF [42]. Similarly, the expression of EMT target genes N-cadherin and ZEB1 [43] were also reduced in ß-catenin-silenced cells (Figure 6B). Interestingly, although not a target gene of the ß-catenin/TCF complex, silencing of ß-catenin in FTC133 markedly decreased Snail expression, whereas the expression of other mesenchymal genes such as fibronectin or vimentin was unchanged (Figure 6B). As EMT is ultimately responsible for tumor cell migration and invasion, we examined the invasion capacity of ß-catenin-silenced FTC133 cells using Matrigel assays. As anticipated, these cells exhibited a significant 3-fold reduction in their invasiveness with respect to the control scramble cells (Figure 6C).

## DISCUSSION

We show that RAS but not BRAF signals through ß-catenin and controls essential oncogenic functions in thyroid cancer cells. HRAS acts predominantly through PI3K/AKT to stabilize ß-catenin via two mechanisms, GSK3ß inhibition and direct ß-catenin phosphorylation at Ser552. PI3K/AKT-mediated ß-catenin activation protects cells from senescence and increases cell proliferation, EMT and cell invasion.

Mutations in ß-catenin have been identified in the most aggressive forms of thyroid cancer, such as poorly differentiated and anaplastic carcinomas. In addition, aberrant expression of ß-catenin and its abnormal cellular localization in the cytoplasm and nucleus occur in tumor cells of differentiated thyroid cancer [14] and, in accordance with our findings, correlates with the presence of oncogenic RAS [14]. The consequences of this dysregulation as well as the functional implications of ß-catenin activating mutations are of great interest due to the potential impact on pathogenesis. Studies on melanoma, a cancer in which ß-catenin is also activated, suggest that the results of Wnt/ ß-catenin signaling activation are complex and likely context-dependent [44]. Similarly, we show here that in the context of precisely defined genetic changes, such as RAS and PTEN loss, ß-catenin is stabilized and expressed in the nucleus and mediates pro-oncogenic effects including evasion from oncogene-induced senescence, EMT and invasion.

An improved understanding of thyroid cancer genetics is beginning to pave the way for the molecular classification of these tumors based on fundamental driving mutations. PTC is a MAPK-driven cancer that has two mutually exclusive drivers with distinct signaling outcomes: BRAF^V600E^ and mutated RAS. Tumors driven by BRAF^V600E^ have high MAPK-signaling and tumors driven by RAS and receptor tyrosine kinase fusions (e.g. RET/PTC) have low MAPK-signaling and have concurrent activation of PI3K/AKT [28] [45]. FTC is considered predominantly driven by PI3K/AKT activation through mutations in RAS, PTEN and RASL1 loss, or by activating mutations in PIK3CA and AKT1 [10, 46]. Overlapping mutations in components of both pathways have been found in the full metastatic thyroid cancer phenotype [47, 48]. The interaction of β-catenin with these oncogenic driver mutations is of great interest as it may determine the fate of the tumor. In transgenic melanoma models, concurrent BRAF^V600E^ and PTEN loss requires ß-catenin stabilization to promote metastasis to lymph nodes and lungs [44]. By contrast, in the presence of wild-type PTEN, BRAF^V600E^ tumors do not metastasize independently of ß-catenin status. Here we show that mutated RAS-driven thyroid tumors need ß-catenin stabilization for tumor progression and this effect requires PI3K/AKT activation and is independent of MAPK signaling.

It has previously been demonstrated that oncogenic RET/PTC rearrangements stabilize ß-catenin, promoting its accumulation in the nucleus and enhancing its activity [25]. Similar to RAS, RET/PTC also results in lower MAPK-signaling and activation of PI3K/AKT [25, 28]. In the presence of RET/PTC rearrangements, the group of Santoro observed that β-catenin stabilization induces a mitogenic effect through the upregulation of TCF/LEF-induced cyclin D1 [49]. By contrast, in the presence of RAS or PTEN loss, we found a strong upregulation of cyclin D1 following ß-catenin silencing. Cyclin D1 upregulation is an established marker of senescence and, accordingly, we found that ß-catenin silencing induced senescence. The reasons for this discrepancy between RET/PTC and RAS/PTEN loss remain incompletely understood. A possible explanation is that in our experiments we achieved a much greater ß-catenin silencing than obtained by Castellone et al. [25] and this may be necessary to induce senescence. Additionally, these differences also emphasize that defining the biological effects of ß-catenin signaling in thyroid cancer may not be possible out of the context of other mutational changes and should help to better classify thyroid cancer into biologically meaningful groups.

Although human thyroid cancer cells with RAS mutations or PTEN loss show strong ß -catenin nuclear localization, there is only a modest activation of the ß-cat/TCF reporter relative to cells without ß-catenin in the nucleus (Figure 2). ß-catenin is a transcriptional regulator that cannot bind DNA by itself and requires binding to other transcription factors to increase the expression of its target genes. Even though TCF/LEF transcription factors are the most studied ß-catenin partners, growing evidence suggest that ß-catenin can associate with other DNA-binding transcription factors, such as FoxO1 [50], the nuclear receptor LRH1 [51] or the thyroid transcription factor Pax8 [23]. Differential expression of those factors in thyroid cells lines could explain the variable ß-catenin transcriptional activity found herein. Future studies will be necessary to identify ß-catenin target genes in the thyroid-specific context.

Another interesting observation is that Cal62 cells, which harbor a K-RAS mutation, present ß-catenin in the membrane, while cells harboring H or N-RAS mutations show nuclear ß-catenin localization. RAS isoforms are functionally non-redundant, mainly due to their different subcellular localization, and it has been shown that K-RAS signals preferentially via BRAF/MAPK while the other isoforms have PI3K as their main effector [52].

How ß-catenin is activated may have important functional implications for its potential use as a therapeutic target. In addition to GSK3ß inhibition, Castellone's work demonstrated RET-induced phosphorylation in ß-catenin at Tyr654 residue. This phosphorylation event is dependent on tyrosine kinase receptors, such as ERB and SRC [53, 54], but has no relationship with AKT activation. By contrast, in a RAS-like genetic context where PI3K/AKT activation is present, phosphorylation of ß-catenin at Ser552 seems to be predominant and is very sensitive to AKT inhibition. Ser552 phosphorylation has been shown to be independent of Wnt factors and MAPK signaling. Thus, strategies to inhibit these signaling pathways may have a very limited effect on ß-catenin activation in RAS-like tumors, whereas AKT inhibition and/or direct inhibition of ß-catenin Ser552 phosphorylation could be a more specific mechanism to inhibit its pro-metastatic effects.

Components of the MAPK and PI3K/AKT signaling pathways are important molecular targets for new cancer therapeutics. For example, selumetinib (a MEK inhibitor) and vemurafenib and dabrafenib (mutant BRAF inhibitors) are clinically beneficial in thyroid cancer patients with advanced metastatic disease. However, resistance to kinase inhibitors targeting components of these signaling pathways is a common phenomenon in patients with advanced radioactive iodine-refractory differentiated thyroid cancer [55]. Understanding how simultaneous activation of additional signaling pathways in a given tumor can impact inhibitor-targeted pathways is essential and may assist in explaining why some patients with a given driver mutation respond differentially to treatment with the same inhibitor. In this context, it is particularly noteworthy that changes in ß-catenin can have such significant effects in RAS-driven tumors.

In conclusion, we provide new insights into the mechanisms by which alterations in ß-catenin signaling impact thyroid cancer formation and progression. Depending on the genetic context (RAS-driven tumors and those where PI3K/AKT is predominantly activated such as PTEN-deficient tumors), ß-catenin is stabilized specifically through phosphorylation at Ser552 and this event protects cells from oncogene-induced senescence and contributes to EMT and cell invasion. ß-catenin targeting approaches could therefore have potential utility in thyroid cancer, particularly in combination with other therapies. Given the interconnectedness of ß-catenin signaling with other oncogenes and tumor suppressor pathways, further studies are clearly necessary to explore the potential value of combinatorial therapies. We believe that our study, together with those reported by Castellone et al. [17] and Gujral et al. [56], will help to identify the genetic factors and biomarkers that can predict responses to treatment with ß-catenin pathway modulators, either alone or in combination with other therapies, and will be an important next step in determining the utility of these potential new therapies.

## MATERIALS AND METHODS

### Cell culture

HeLa and HEK293T cell lines were grown in Dulbecco's modified Eagle's medium (DMEM) supplemented with 5% and 10% fetal bovine serum (FBS), respectively. PC-BRAF and PC-HRAS were derived from PCCl3 rat thyroid follicular cells [57] to obtain doxycycline-inducible expression of BRAF^V600E^ or HRAS^V12^ [58, 59]. Cells were grown in Coon's modified Ham's F-12 medium supplemented with a six-hormone mixture (1 nM TSH, 10 μg/ml insulin, 10 ng/ml somatostatin, 5 μg/ml transferrin, 10 nM hydrocortisone, and 10 ng/ml glycyl-L-histidyl-L-lysine acetate) and 5% donor calf serum (complete). To avoid the effects of TSH and IGF1 on the Wnt signaling pathway [23], PC-HRAS and PC-BRAF cells were transferred to Coon's starvation medium supplemented with 0.2% BSA plus hormones, but without TSH and insulin for 48 h. Cells were treated with 1 μg/mL doxycycline for the indicated time periods to induce BRAF or HRAS expression.

Thyroid cancer cell lines were from the following sources: C643, Hth7, Hth83 and SW1736 were originally from Dr. N.E. Heldin (University of Uppsala, Uppsala, Sweden); BCPAP was from Dr. M. Santoro (University of Federico II, Naples, Italy); FTC133 and Nthy-Ori-3.1 were obtained from the European Collection of Cell cultures; WRO-82-1 was from Dr. G. J. F. Juillard (University of California-Los Angeles School of Medicine, Los Angeles, CA); TPC1 was provided by Dr. A.P. Dackiw (Johns Hopkins University, Baltimore, Maryland); KTC-1 was provided by Junichi Kurebayashi (Kawasaki Medical School, Japan); CAL62, FRO and 8505c were obtained from the Leibniz-Institut DSMZ-German Collection of Microorganisms and Cell Cultures and T238 was from Dr. Lucia Roque (Portuguese Cancer Institute, Lisbon, Portugal). Cell lines were authenticated every 6 months by short tandem repeat (STR) profiles using the Applied Biosystems Identifier kit in the Genomic Facility at the Instituto de Investigaciones Biomédicas (Madrid, Spain). Cell lines were grown at 37°C in RPMI 1640 with 10% FBS, except FTC133, which was cultured in DMEM. The following inhibitors were added to cells at the indicated concentrations: 10 μM UO126 (Calbiochem), 10 μM AKT-i-VIII, 10 μM XAV939 and 10 μM bortezomib (Sigma).

### Luciferase reporter assay

To measure ß-catenin transcriptional activity, cells were transfected with Super8xTop- and Fop-Luc luciferase vectors, which contain 8 optimized and 8 mutated TCF-binding sites, respectively (both kind gifts of R.T. Moon) [60]. To measure ERK activity, cells were transfected with the reporter pGE51-luc and a vector encoding the transactivation domain of Elk-1 (amino acids 307 to 428) fused to the GAL4 DNA-binding domain [61]. Additionally, a pRL-CMV Renilla luciferase construct (Promega) was co-transfected to monitor transfection efficiency. HRAS^V12^ and BRAF^V600E^ expression vectors were used in transfection experiments as described [62].

Thyroid cancer cells were grown in DMEM or RPMI and transfected with JetPEI transfection reagent (Polyplus). Twenty-four hours after transfection, cells were treated with the indicated compounds for 24 h and harvested for luciferase measurement (Dual-Luciferase kit, Promega). PC-BRAF and PC-HRAS cells were transfected in Coon's complete medium using JetPEI or by calcium phosphate coprecipitation [63], grown for 48 h in starvation medium and treated with doxycycline for the indicated time periods. HeLa cells were grown in DMEM complete medium, transfected by calcium phosphate coprecipitation, and harvested 48 h later. One microgram of promoter construct was co-transfected with 1 μg of the indicated expression vector. The amount of DNA in each transfection was kept constant by the addition of an appropriate amount of empty expression vector, pcDNA3.1. To normalize transfection efficiency, 50 ng of pRL-CMV vector was added in all cases. Additionally, in the Top/Fop assays, Top activity was normalized with Fop activity to ensure that Top Flash values were dependent on the Lef1/Tcf-binding sites in the promoter. All transfection experiments were performed in triplicate and carried out at least three times.

### Western blotting

Total proteins were lysed in a buffer containing 1% NP-40, 0.1% SDS, 0.5% deoxycholic acid, supplemented with a protease inhibitor cocktail (Roche). Nuclear and cytoplasmic proteins were extracted as described [64]. Protein concentration was measured according to the method of Bradford [65] using an assay from Bio-Rad Laboratories. Equal amounts of protein were diluted in loading buffer and heated at 95°C for 5 min. Samples were separated by SDS-PAGE, transferred onto nitrocellulose membranes, blocked and incubated overnight with primary antibodies diluted in PBS 0.1% Tween 20 containing 5% w/v nonfat dry milk. Antibody binding was revealed with horseradish peroxidase (HRP)-conjugated secondary antibodies and immunoreactive proteins were visualized by enhanced chemiluminescence (Thermo Scientific). Protein expression levels were quantified using ImageJ software (NIH) and normalized to their loading controls. Antibodies to streptavidin-(HRP), AKT1/2, pAKT (S473), ß-actin, ß-catenin, cyclin D1, p27, pERK (Y204), ERK1/2 and tubulin were obtained from Santa Cruz Biotechnology. Antibodies to phospho-β-catenin (S33/37/T41 and S552), CTCF and phosph-GSK3ß (S9) were from Cell Signaling Technology Antibodies to E-cadherin and GSK3ß were from BD Biosciences.

### Semi-quantitative RT–PCR

RNA was extracted using Trizol (Sigma) and equal amounts of RNA were added to a reverse-transcriptase reaction mix (m-MLV, Promega). Real time PCR was performed on an Mx3000 platform (Agilent) using the FastStart Universal Probe Master kit (ROX, Roche) and run for 40 cycles. Specificity of the reactions was determined by subsequent melting curve analysis. Stratagene analysis software was used to remove background fluorescence [66]. The number of cycles needed to reach the crossing point for each sample was used to calculate the amount of each product using the 2^−Δ Δ Ct^ method. The levels of PCR product were expressed as a function of actin. The primers used are listed in Supplementary Table 1.

### Immunofluorescence

Cells were seeded onto coverslips and treated as indicated. Unfixed tumors were embedded in OCT (Tissue tek). Frozen tissue was sectioned (10 μm, Leica cryostat) and mounted on SuperFrost Plus slides (Fisher). Cells and frozen sections were fixed with 4% paraformaldehyde in PBS pH 7.4 for 10 min, rinsed with PBS, permeabilized with 0.5% Triton X-100, and blocked with 1% BSA. After over-night incubation with primary ß-catenin (C-18) or phospho-Ser552 ß-catenin antibodies [67] (provided by Dr. Linheng Li), samples were rinsed with PBS and incubated with Alexa 488 or Texas Red 546 secondary antibodies for one hour, mounted with ProLong^®^ Gold antifade reagent with DAPI (Invitrogen) and observed under a confocal microscope with a 63× magnification objective (Leica).

### Lentivirus production and cell infection

Stable thyroid cancer cell lines were generated using lentiviral expression vectors. For the generation of β-catenin-silenced cells (shβ-cat) the vector encoded an shRNA against human βcatenin (pLKO.1-shbcat; Open Biosystem clone TRCN0000003845). Control cells (shScramble) were infected with virus containing a non-coding control DNA (pGIPZ-shscramble, Open Biosystem). Cells expressing a dominant negative form of TCF (dnTCF4) were generated using the vector EdTP (vector was from Addgene and is described in [36]). VSV-G pseudotyped lentivirus production was performed as previously described [23]. Forty-eight hours after infection, puromycin-resistant cells were selected with 1 μg/ml of puromycin (Sigma).

### Cell proliferation and BrdU incorporation assay

To determine cell proliferation cells were plated at 2 × 10^5^ cell per well in 24-well plates. Cells were fixed at the indicated times with 1% glutaraldehyde and stained with crystal violet. After extensive washing, crystal violet was resolubilized in 1% acetic acid and quantified at 595 nm as an indirect measure of cell number. DNA synthesis was determined using the Cell Proliferation ELISA BrdU Assay (Roche Molecular Biochemicals). Briefly, cells were seeded in 96-well plates (5 × 10^3^ cells/well). Twenty-four hours later, cells were pulse labeled for 2 h with 10 μM BrdU, and measurements were carried on an ELISA reader at 450 nm. Experiments were performed three times in triplicate.

### Senescence assay (SA-ß-gal staining)

Thyroid cells were infected and selected and plated on 12-well plates (7 × 10^5^ cells/well). Twenty-four hours later, cells were fixed and assayed for SA-ß-gal activity using the Senescence Detection kit (BioVision). To obtain the percentage of SA-ß-gal positive cells, four images of each condition were captured using 10× magnification and quantified. Experiments were repeated at least three times.

### Invasion assay

Invasion was measured in Transwell cell culture chambers using BD BioCoat Matrigel Invasion Chambers (BD Bioscience). Infected FTC133 cells (2.5 × 10^4^) were placed on the upper chamber in DMEM plus 0.2% FBS. The lower chamber contained DMEM supplemented with 15% FBS. Cells were allowed to invade for 24 h at 37°C and 5% CO_2_. Nonmigrated cells on the upper chamber were removed with a cotton swab; filters were fixed in 4% paraformaldehyde and stained using the Diff-Quik staining kit (BD Biosciences). The total number of cells migrated to the lower surface was counted. The experiments were repeated three times.

### Xenograft studies in mice

Protocols for animal handling were approved by the Institutional Animal Care Committee, following the rules of the European Union and the National Institutes of Health. Animals were housed in temperature-controlled rooms (22 ± 2°C) with 12-h light/dark cycles (lights on at 07:00 h) and had free access to food and water. For xenograft studies 9 × 10^6^ FTC133 shScramble or shβ-cat cells were subcutaneously injected into two-month-old female NOD-SCID mice. Tumors were measured every 4 days and the tumor volume was calculated as π*l*w/6, where l = length in mm and w = width in mm.

### Statistical Analysis

Results are presented as mean fold induction ± SEM from at least three independent biological experiments. Student's two-tailed *t*-test was used to assess differences between measurements. Differences were considered statistically significant if *p* < 0.05.

## SUPPLEMENTARY MATERIALS FIGURE AND TABLE


