# Supplementary material for: ß-catenin signaling is required for RAS-driven thyroid cancer through PI3K activation
Source: Oncotarget. 2016 Jun 30;7(31):49435–49. doi: 10.18632/oncotarget.10356 (PMC5226519; doi:10.18632/oncotarget.10356)
Supplement: Supplementary file 1 [file oncotarget-07-49435-s001.pdf]

# ß-catenin signaling is required for RAS-driven thyroid cancer through PI3K activation

## Supplementary Materials

### Supplementary Table S1: Primers used for RT-QPCR

| Gene | Forward primer | Reverse primer | Size (bp) |
|---|---|---|---|
| p21 | 5′CTTGTACCCTTGTGCCTCG3′ | 5′TTAGGGCTTCCTCTTGGAGA3′ | 149 |
| β-catenin | 5′CCATCTGTGCTCTTCGTCATCT3′ | 5′GCAGTCTCATTCCAAGCCAT3′ | 691 |
| E-cadherin | 5′AAGGGCTTGGATTTTGAGGCCA3′ | 5′GTGCTGTTCTTCACAGGCTC3′ | 362 |
| Slug | 5′TGGTTGCTTCAAGGACACAT3′ | 5′GTTGCAGTGAGGGCAAAGAA3′ | 66 |
| TWIST1 | 5′CCGGAGACCTAGATGTCATTG3′ | 5′ATGCAGAGGTGTGAGGATGG3′ | 234 |
| SNAIL1 | 5′ACCCACACTGGCGAGAAG3′ | 5′GAGAAGGATGTGGGGTCCTT3′ | 281 |
| ZEB1 | 5′GCTGACCAGAACAGTGTT3′ | 5′CAGAGTCATTCTGATCCTC3′ | 211 |
| Fibronectin | 5′CCCTTACAGTTCAGGGTTCC3′ | 5′TTCAAGCCTTCGTTGACAGA3′ | 164 |
| Vimentin | 5′GACAATGCGTCTCTGGCACGT3′ | 5′TCTTCTGCCTCCTGCAGGTTCTT3′ | 236 |
| N-cadherin | 5′TGCCATGACGTTTTATGGTG3′ | 5′TGGTTTGACCACGGTGACTA3′ | 199 |

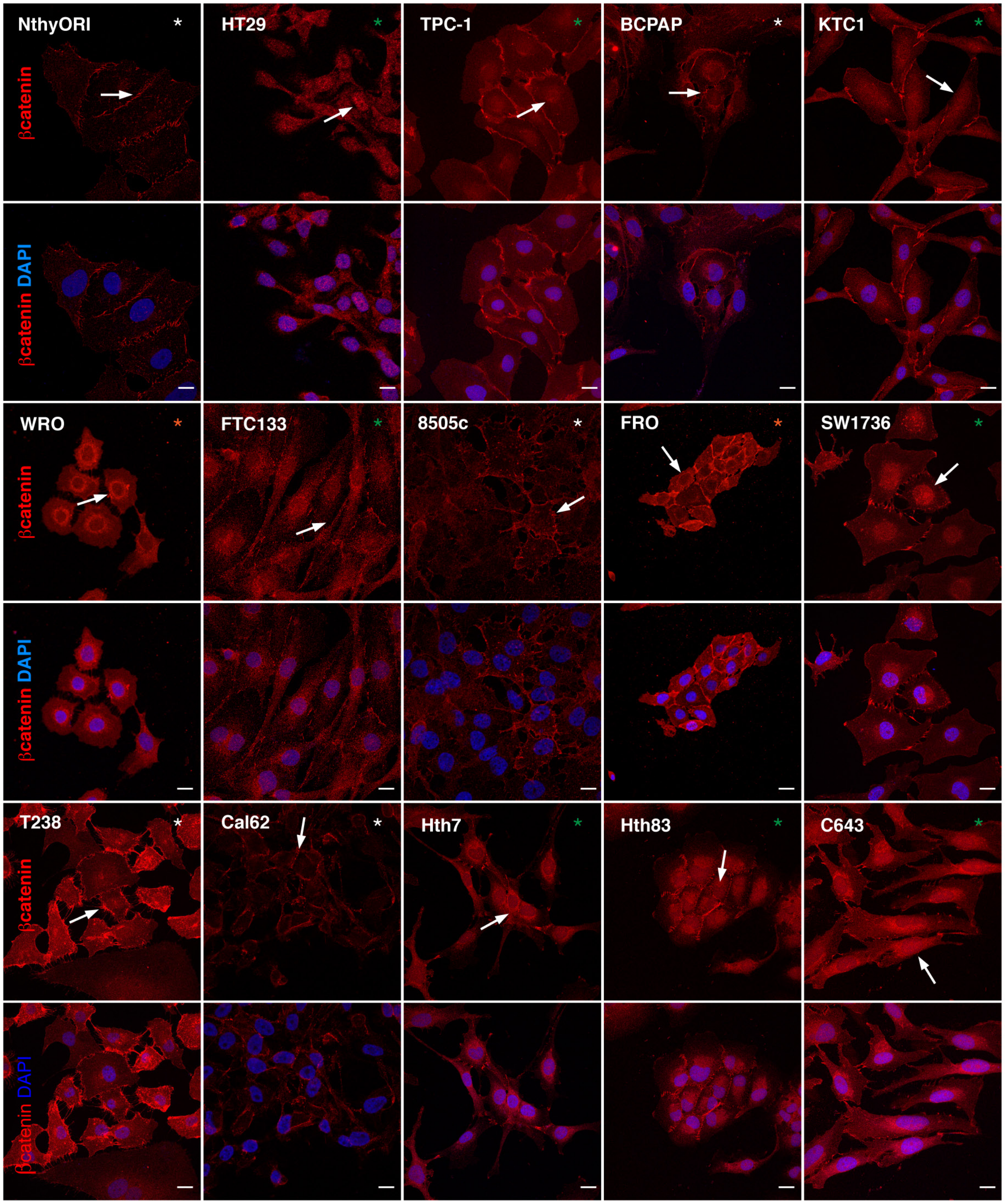


**Supplementary Figure S1: ß-catenin cellular localization in human thyroid cancer cell lines.** Cells were grown in cover-slips, fixed and stained with a ß-catenin antibody (red). Nuclei were stained with DAPI (blue). Scale bar 10 µm. Asterisks indicate membrane (white), perinuclear (orange) or nuclear localization (green). The arrows show representative ß-catenin staining.
